# Anti-Inflammatory, Thrombolytic and Hair-Growth Promoting Activity of the *n*-Hexane Fraction of the Methanol Extract of *Leea indica* Leaves

**DOI:** 10.3390/plants10061081

**Published:** 2021-05-27

**Authors:** Shahenur Alam Sakib, Abu Montakim Tareq, Ameerul Islam, Ahmed Rakib, Mohammad Nazmul Islam, Mohammad Arafat Uddin, Md. Masudur Rahman, Veronique Seidel, Talha Bin Emran

**Affiliations:** 1 Department of Pharmacy, International Islamic University Chittagong, Kumira, Chittagong 4318, Bangladesh; sakibhasaniiuc@gmail.com (S.A.S.); montakim0.abu@gmail.com (A.M.T.); iamirul7@gmail.com (A.I.); sayeadiiuc@gmail.com (M.N.I.); arfatmasud09@gmail.com (M.A.U.); 2 Department of Pharmacy, Faculty of Biological Sciences, University of Chittagong, Chittagong 4331, Bangladesh; rakib.pharmacy.cu@gmail.com; 3 Natural Products Research Laboratory, Strathclyde Institute of Pharmacy and Biomedical Sciences, University of Strathclyde, Glasgow G4 0RE, UK; 4 Department of Pharmacy, BGC Trust University Bangladesh, Chittagong 4381, Bangladesh

**Keywords:** anti-inflammatory activity, thrombolytic activity, hair growth-promoting activity, *Leea indica*

## Abstract

The anti-inflammatory, thrombolytic, and hair growth-promoting activity of the *n*-hexane fraction from the methanol extract of *Leea indica* (NFLI) leaves was investigated. NFLI showed significant inhibition of hemolysis and protein denaturation, and exhibited a concentration-dependent thrombolytic activity. When applied topically to mice at concentrations of 10, 1, 0.1%, NFLI demonstrated a significant increase in average hair length (*p* < 0.001) compared with untreated animals. NFLI (1% concentration) exhibited the highest percentage of hair regrowth on day 7, 14 and 21 (81.24, 65.60, and 62.5%, respectively). An in silico study was further conducted to predict the binding affinity of phytochemicals previously reported in *L. indica* towards PGD_2_ synthase (PDB ID: 2VD1), an enzyme that catalyses the isomerisation of prostaglandin H_2_ to PGD_2_ which is involved in hair loss. Phthalic acid, farnesol, *n*-tricosane, *n*-tetracosane, and *n*-heptacosane showed the best ligand efficiencies towards PGD_2_ synthase and their intermolecular interactions were visualised using BIOVIA Discovery Studio Visualizer. Our results indicate that *L. indica* could represent a promising natural alternative to tackle alopecia.

## 1. Introduction

*Leea indica* (Burm.f.) Merr. (Vitaceae) is an evergreen shrub native to India, China, Bhutan, Malaysia, Thailand, and Bangladesh. The plant is employed in traditional medicine for headaches, body pains, and skin problems [1,2]. Its roots are used as sudorific, antispasmodic, antidiarrhoeal, antidysenteric, and to treat cardiac and skin diseases [3]. The leaves are used to treat diabetes, obstetric diseases, and vertigo [3,4,5,6]. *Leea indica* leaves contain long-chain hydrocarbons, phthalate derivatives, palmitic acid, gallic and ellagic acid derivatives, solanesol, phytosterols, triterpenes, catechins, condensed tannins, flavonoids, coumarins, megastigmanes, and oxylipins [7,8,9].

Crude extracts of *L. indica,* and some of its constituents, have demonstrated a range of biological effects [9,10,11,12,13,14,15,16,17,18,19,20,21,22,23,24,25,26,27]. Previous pharmacological studies on the methanol extract of *L. indica* leaves revealed that it exhibited sedative and anxiolytic [10], lipase inhibitory [12], anticancer [19], antimicrobial and antidiarrheal [23], antihyperglycemic [24], antioxidant [13,19,25], larvicidal [26], and antimalarial activity [25]. The primary aim of the present study was to investigate the anti-inflammatory, thrombolytic activity and hair growth-promoting activity of the *n*-hexane fraction of the methanol extract of *L. indica* (NFLI) leaves. In addition, we also sought to predict the binding affinities and ligand efficiency indices of 16 phytochemicals previously reported in a non-polar fraction of the methanol extract of *L. indica* leaves [7] towards PGD_2_ synthase, a target enzyme for the treatment of hair loss [28].

## 2. Results

### 2.1. Anti-Inflammatory Activity

#### 2.1.1. Membrane Stabilisation Assay

**Figure 1:**
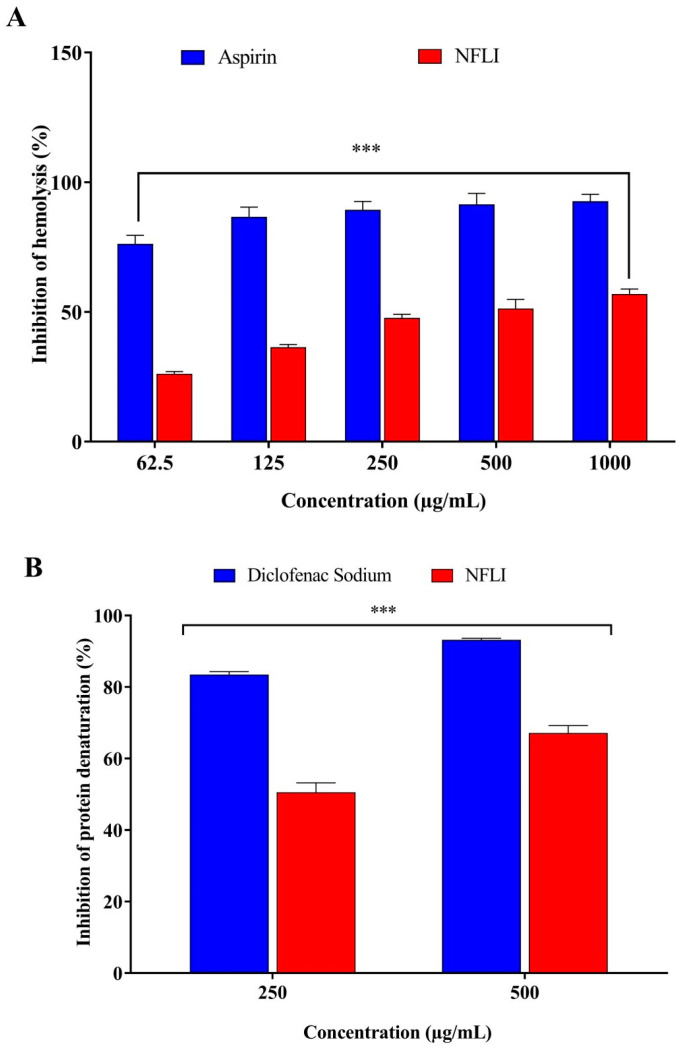
Anti-inflammatory activity of *n*-hexane fraction of *L. indica* (NFLI). (**A**) Membrane stabilisation effect of the *n*-hexane fraction of *L. indica* (NFLI) compared with aspirin. (**B**) Percentage inhibition of protein denaturation of the *n*-hexane fraction of *L. indica* (NFLI) compared with diclofenac. Results are expressed as mean ± SEM, with *** *p* < 0.001 considered significantly different to the positive control group following one-way ANOVA (Dunnett’s test).

NFLI showed a concentration-dependent activity with the 1000 μg/mL concentration showing the highest inhibition (56.92 ± 1.90%). At the same concentration, the standard drug aspirin showed 92.63 ± 2.71% inhibition of hemolysis (Figure 1A).

#### 2.1.2. Inhibition of Protein Denaturation

The highest inhibition was observed for NFLI at 500 μg/mL (67.11 ± 2.11%). The standard drug diclofenac showed inhibition of 93.18 ± 0.42% (Figure 1B).

### 2.2. Thrombolytic Activity

**Figure 2:**
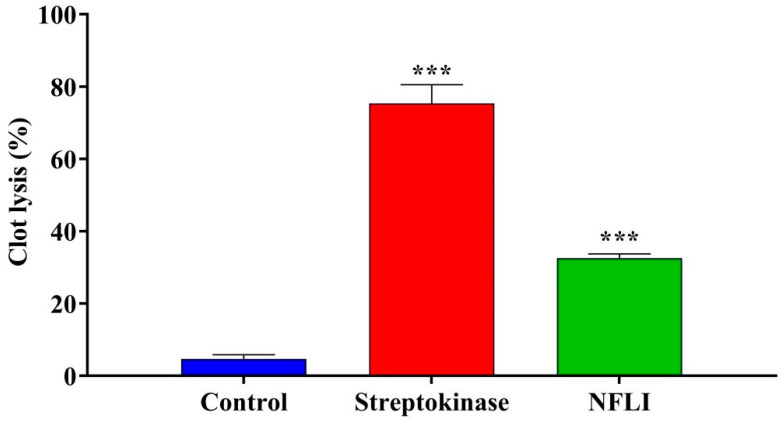
Thrombolytic activity of the *n*-hexane fraction of *L. indica* (NFLI) compared with streptokinase. Results are expressed as mean ± SEM, with *** *p* < 0.001 considered significantly different to the negative control group following one-way ANOVA (Dunnett’s test).

NFLI (10 mg/mL, 0.1% concentration) and streptokinase exhibited significant (*p* < 0.001) thrombolytic activity compared to the negative control group (32.58 ± 1.18% and 75.35 ± 5.21%, respectively) (Figure 2).

### 2.3. Hair-Growth Promoting Activity

**Figure 3:**
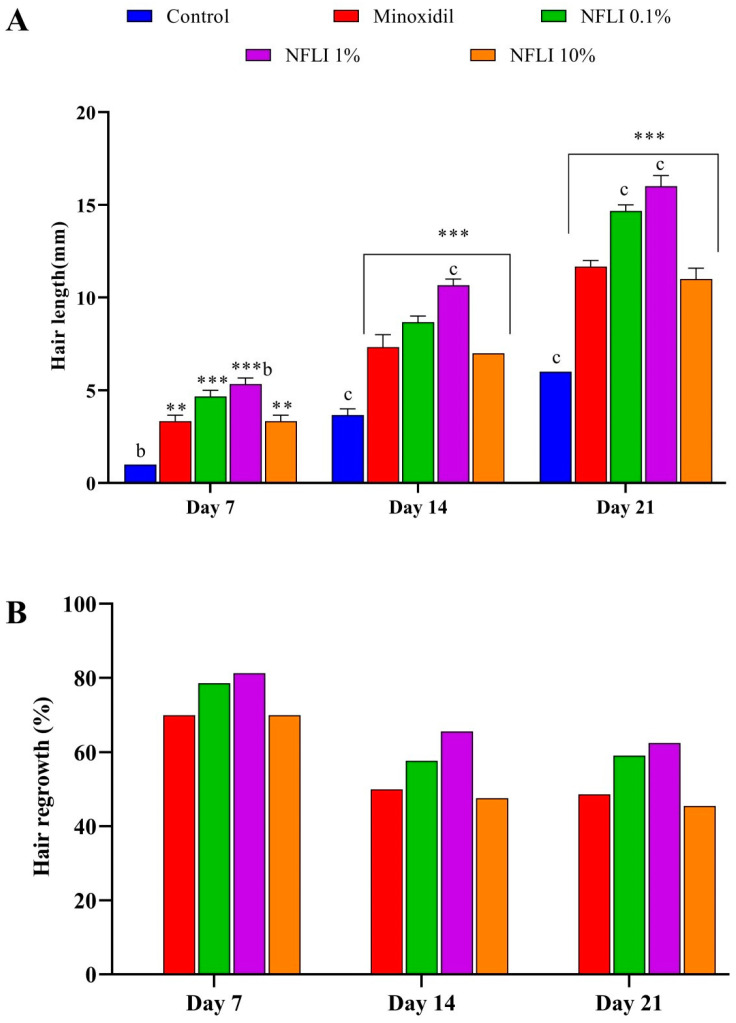
Average hair length (**A**) and percentage of hair regrowth (**B**) recorded in different animal groups at day 7, 14, and 21. Results are expressed as mean ± SEM. Two-way ANOVA (followed by Tukey’s test) was used to analyse the statistical significance of the data obtained, with *p* < 0.05 considered significantly different. ** (*p <* 0.01) and *** (*p <* 0.001) denote statistical significance versus the negative control (1% Tween 80 in water). ^b^ (*p <* 0.01) and ^c^ (*p <* 0.001) denote statistical significance versus the positive control (minoxidil).

**Figure 4:**
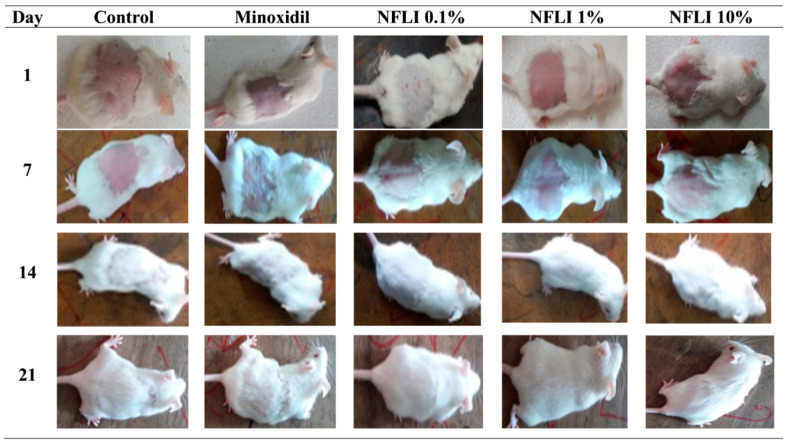
Hair growth-promoting effects of the *n*-hexane fraction of *L. indica* (NFLI) and minoxidil in mice after 1, 7, 14, and 21 days.

The average hair length recorded at day 7, 14, and 21 increased significantly (*p* < 0.001) following treatment with NFLI at all concentrations in comparison with the negative control group (Figure 3A). In addition, NFLI at the 1% concentration showed the highest percentage of hair regrowth at day 7, 14, and 21 (81.24, 65.60, and 62.5%, respectively) (Figure 3B). This was higher than the standard drug minoxidil (69.97, 49.93, and 48.59%, respectively). Additionally, NFLI at the 0.1% concentration exhibited 78.59, 57.67, and 59.10% of hair regrowth at day 7, 14, and 21. NFLI at the 1% concentration demonstrated significant (*p* < 0.001) hair length growth compared to the minoxidil (positive control). The effects of topical applications of NFLI and minoxidil to mice after 1, 7, 14, and 21 days are illustrated in Figure 4.

### 2.4. Molecular Docking of 16 Phytochemicals Previously Reported in L. indica against PGD_2_ Synthase

**Figure 5:**
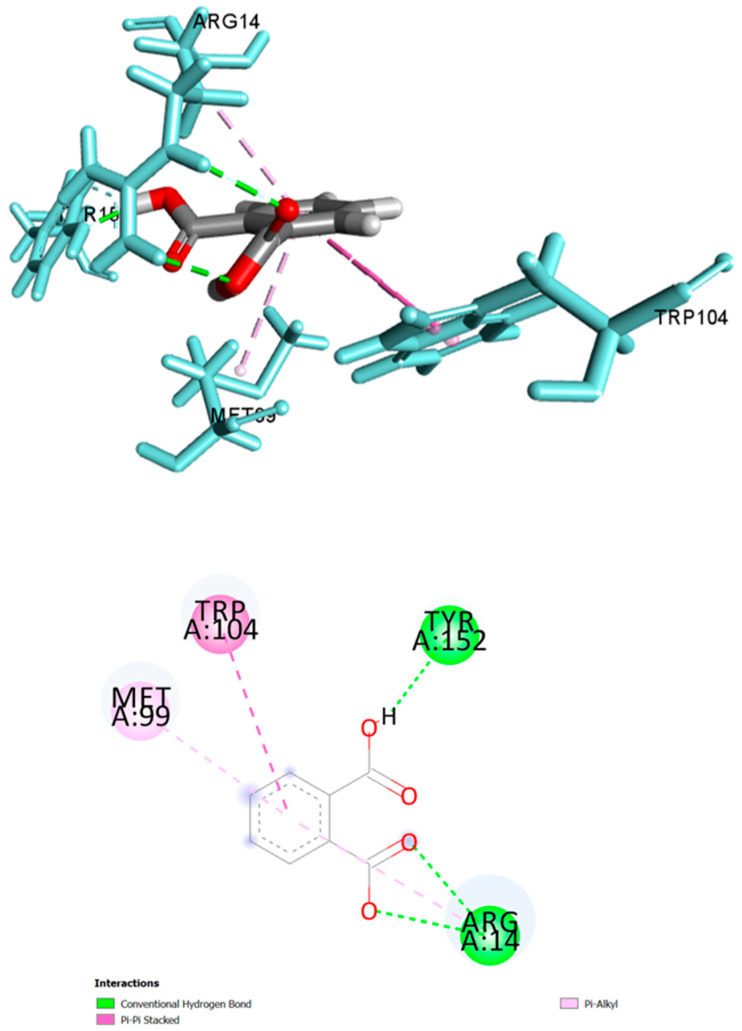
Molecular docking study of *L. indica* phytochemicals. (**A**) Docked pose of phthalic acid in the PGD_2_ synthase binding site showing molecular interactions—hydrogen and hydrophobic bonds as green and pink/purple dashed lines, respectively; (**B**) 2D plot of interactions between phthalic acid and key residues of PGD_2_ synthase generated by BIOVIA Discovery Studio visualizer.

Molecular docking was used to predict the binding affinities and ligand efficiency indices of 16 phytochemicals previously reported in the petroleum ether fraction of the methanol extract of *L. indica* leaves [7] towards PGD_2_ synthase. Owing to the very similar polarity between the extraction solvent used in this previous study and our present investigation, it is probable that such phytoconstituents are present in NFLI. Solanesol showed the highest docking score (−7.133 kcal/mol), followed by β-sitosterol (−6.191 kcal/mol), phthalic acid (−6.078 kcal/mol), and lycopersen (−5.672 kcal/mol). The positive control glutathione had a docking score of −5.278 kcal/mol. Palmitic acid, *n*-heptadecane, *n*-octadecane, *n*-eicosane, 1-eicosanol, and 17-pentatriacontene showed very low docking scores and were not analysed further. Among the remaining 10 compounds, the best ligand efficiencies towards PGD_2_ synthase were obtained for phthalic acid (3.49), farnesol (3.08), *n*-tricosane (2.24), *n*-tetracosane (1.99), and *n*-heptacosane (1.90) (Table 1). The molecular interactions of these compounds are detailed in Table 1. Phthalic acid strongly interacted with PGD_2_ synthase via two hydrogen bonds (contact distances < 2.5 Å) to Arg14 and one to Tyr152 as well as hydrophobic bonds with Trp104, Met99, and Arg14 (Table 2 and Figure 5).

## 3. Discussion

This study was conducted to investigate the anti-inflammatory, thrombolytic, and hair growth-promoting activity of NFLI. We observed that NFLI showed a significant dose-dependent inhibition of hemolysis and protein denaturation compared to two non-steroidal anti-inflammatory drugs. It is interesting to note that peripheral antinociceptive activity (which may be associated with reduced inflammation) has been reported for an ethanol extract of *L. indica* leaves [27]. This supports the traditional use of *L. indica* to alleviate pain. Further in vivo investigations are required to confirm this anti-inflammatory effect and provide new prospects for the discovery of anti-inflammatory drugs of natural origin.

We also showed that NFLI had significant thrombolytic activity, in agreement with a previous study on the ethanol extract of *L. indica* leaves [15]. Further investigations are required to identify the active substance(s) which may provide new templates for the discovery of safer drugs to treat deep venous thrombosis, pulmonary embolism, and myocardial/cerebral infarctions [29].

We observed that NFLI had hair growth-promoting activity. To the best of our knowledge, this has never been reported. Vast sums of money are spent annually on hair-growth products by both men and women who suffer from hair loss. Composed of terminally differentiated and dead keratinocytes, hair plays a role, as a protective and sensory mini-organ, in thermoregulation and sexual attractiveness [30]. The process of hair growth is divided into the anagen (growth), telogen (resting), catagen (regression), and exogen (shedding) phases [31,32,33]. Any dysregulation of this normal cycle leads to alopecia. The latter can be caused by hormonal imbalance, nutritional deficiency, cancer chemotherapy, and/or excessive stress. It is prevalent in individuals with a genetic predisposition (e.g., androgenetic alopecia-AGA) [34,35,36]. Oral medicines used to treat alopecia present various adverse side effects and topical formulations can cause local irritation, therefore alternative treatment options are warranted [37,38,39,40,41].

Hair loss, particularly in patients with androgenetic alopecia, has been linked to the presence of high levels of prostaglandin D_2_ (PGD_2_) [42,43]. One approach to reduce the amount of PGD_2_ involves the inhibition of prostaglandin D_2_ synthase, an enzyme that catalyses the isomerisation of prostaglandin H_2_ to PGD_2_ [28,44]. As previous reports had indicated the presence of various classes of phytochemicals (hydrocarbons, terpenes, and aromatic and fatty acids) in a petroleum ether fraction—similar in polarity to NFLI—of the methanol extract of *L. indica* [7], we decided to employ a molecular docking approach to predict the binding affinity of these phytochemicals towards PGD_2_ synthase. This enzyme has been previously used as a target for the search of natural products with hair growth-promoting activity [28]. Five phytochemicals showed greater ligand efficiency than the positive control glutathione. Among them, phthalic acid demonstrated the highest ligand efficiency and interacted with Arg14 and Tyr152 (H-bonds), and Arg14, Met99, and Trp104 (hydrophobic bonds) identified as key residues of the catalytic site of PGD_2_ synthase [44,45,46]. Further work is required to test the hair growth-promoting effect of phthalic acid, which unlike phthalates, has a low toxicity profile [47]. Additionally, it is possible that other phytochemicals identified in the non-polar fraction of the methanol extract of *L. indica* leaves may contribute via other mechanisms to the observed activity. This includes β-sitosterol, which is already known to inhibit 5α-reductase and lower dihydrotestosterone levels associated with balding scalp skin in AGA [36,48,49]. Although our study was not pursued beyond 21 days (topical treatments for alopecia are used for several months), our results contribute to advancing knowledge in the field of plants/herbal medicines as potential hair-growth promoters [50,51] and suggest that NFLI could be a natural alternative to current hair-loss treatments.

## 4. Materials and Methods

### 4.1. Chemicals and Reagents

The standard drug minoxidil was purchased from Renata Ltd. (Bangladesh). Streptokinase and diclofenac sodium were procured from Beacon Pharmaceuticals Ltd. and Square Pharmaceuticals Ltd. (Bangladesh), respectively. Ascorbic acid and quercetin were purchased from BDH Chemicals Ltd. (Poole, UK). Gallic acid, 1,1-diphenyl-2-picrylhydrazyl radical (DPPH), trichloro-acetic acid, aspirin, and the Folin-Ciocalteau reagent (FCR) were procured from Sigma Chemicals Co. (St. Louis, MO, USA). All other analytical grade chemicals were obtained through Taj Scientific Ltd. (Chittagong, Bangladesh).

### 4.2. Extraction of Plant Material

*L. indica* flowering plants (5 kg) were collected in January 2020 from Kaptai, Chittagong (Bangladesh) and were authenticated by Professor Dr. Shaikh Bokhtear Uddin at the Department of Botany, University of Chittagong (Accession No. 36078). The leaves were dried separately in the shade, ground to a coarse powder, and stored in an airtight container at room temperature (25 ± 2 °C). The dried powdered leaves (400 g) were soaked in methanol (1L) with shaking on mechanical shaker (100 rpm). After seven days, the plant extract was filtered with Whatman #1 filter paper using a Buchner funnel. The resulting filtrate was concentrated under reduced pressure at <40 °C to obtain a crude methanolic extract (36.48 g, 9.12% *w*/*w*). The latter was further fractionated using n-hexane (100 mL × 3) to afford the n-hexane fraction of *L. indica* (NFLI) (2 g) which was stored at 4 °C prior to biological work [52].

### 4.3. Experimental Animals

Swiss Albino mice of both sexes, weighing about 30–40 g, were procured from the Bangladesh Council of Scientific and Industrial Research (BCSIR), Chittagong, and were housed at temperature (25 ± 2 °C), relative humidity (5–60%), and 12 h light/dark cycles during an acclimatisation period of 10 days. The animals were provided with standard laboratory food (provided by BCSIR) and distilled water ad libitum. All experiments were conducted in noiseless conditions. The study was approved by the Institutional Animal Ethical Committee, Department of Pharmacy, International Islamic University (Chittagong, Bangladesh) according to governmental guidelines under the reference of Pharm-107/11-17.

### 4.4. Anti-Inflammatory Activity

#### 4.4.1. Membrane Stabilisation Assay

##### Preparation of Erythrocyte Suspension

The anti-inflammatory activity of NFLI was determined based on a previously described method [53] with slight modifications. Here, blood (5 mL) was withdrawn from a healthy human volunteer and mixed with Alsever’s solution (2% dextrose, 0.8% Na-citrate, 0.5% citric acid, and 0.42% sodium chloride). The solution was centrifuged at 3000 rpm for 20 min. Then, the blood cells were washed with normal saline (0.85%) and a 10% (*v*/*v*) suspension was prepared.

##### Hypotonicity-Induced Human Red Blood Cell Hemolysis

Serially-diluted concentrations of NFLI (62.5–1000 µg/mL) and aspirin were used to evaluate the effects of hemolysis on human red blood cells (RBC). Test samples consisted of 0.5 mL of RBC suspension mixed with 2 mL of NaCl (50 mM) and 1 mL of sodium phosphate buffer saline (10 mM, pH 7.4) containing either NFLI or aspirin. The control sample was prepared as above but without NFLI or aspirin. All prepared solutions were incubated for 30 min at 37 °C and then centrifuged at 3000 rpm for 20 min. The absorbance was read at 540 nm. The percentage inhibition of hemolysis was calculated using the following equation:

$$\mathrm{Inhibition}\;\mathrm{of}\;\mathrm{hemolysis}\;\left(\%\right)=\frac{\mathrm{Absorbance}\;\mathrm{of}\;\mathrm{control}-\;\mathrm{Absorbance}\;\mathrm{of}\;\mathrm{test}\;\mathrm{sample}\;}{\mathrm{Absorbance}\;\mathrm{of}\;\mathrm{control}\;}\times 100$$

#### 4.4.2. Protein Denaturation Assay

This was assayed based on a previously described method [54]. Briefly, 5 mL of a reaction mixture consisting of egg albumin (0.2 mL), phosphate buffered saline pH 6.4 (2.8 mL), and *L. indica* extract or distilled water (control) (2 mL) was prepared. Diclofenac sodium was used as the control. Two different concentrations of extract and diclofenac sodium (500 µg/mL, 250 µg/mL) were prepared. After that, each 5 mL of solution of extract and standard drug solution were added in their respective test tubes. The mixture was incubated at 57 °C for 20 min and then heated at 70 °C for 5 min. After cooling, the absorbance of the solutions was measured at 660 nm against a blank solution (solution excluding the extract/standard). The percentage inhibition of protein denaturation was calculated using the formula below:

$$\mathrm{Inhibition}\;\mathrm{of}\;\mathrm{protein}\;\mathrm{denaturation}\;\left(\%\right)=\frac{\mathrm{Absorbance}\;\mathrm{of}\;\mathrm{control}-\mathrm{Absorbance}\;\mathrm{of}\;\mathrm{test}\;\mathrm{sample}\;}{\mathrm{Absorbance}\;\mathrm{of}\;\mathrm{control}\;}\times 100$$

### 4.5. Thrombolytic Activity

This was tested as described previously [55]. Blood (5 mL) was withdrawn from healthy human volunteers (*n* = 5) (non-smoker, no history of taking medications in last week). The blood was distributed in 0.5 mL/tube and incubated (37 °C, 45 min) to form the clot. After forming the clot, the obtained serum was completely removed without disturbing the clot. Again, the tube was reweighed and 100 µL of extract (10 mg/mL), 100 µL of streptokinase, and 100 µL of distilled water individually added to the tube. Then, the tube was again incubated for 90 min at 37 °C and the clot formation observed. After incubation, the tube was again weighed and the clot lysis calculated. The percentage of clot lysis was measured using the following equation: % of clot lysis = (weight of released clot/clot weight) × 100.

### 4.6. Hair Growth-Promoting Activity

#### 4.6.1. Preparation of Samples for Topical Application

NFLI (0.1 g) was dissolved in 1% Tween 80 in water (1 mL) to afford a 10% (*w/v*) stock concentration and was used for topical application in the hair-growth promoting study. Further 1:10 and 1:100 dilutions of this stock were made in water to obtain 1% (*w/v*) and a 0.1% (*w*/*v*) concentrations, respectively.

#### 4.6.2. Hair Growth-Promoting Assay

The animals were randomly divided into five groups consisting of three mice each (15 mice in total). A 4 cm^2^ area of hair from the dorsal portion of all animals was shaved with surgical hair removal cream. Minoxidil (100 µL) and NFLI (10 µL of 0.1%, 1%, and 10% concentrations) were applied once a day to the denuded area of the positive control and the test groups, respectively. The negative control group received no treatment. Hair regrowth was observed visually and recorded 7, 14, and 21 days after the initial topical application. The weights of all animals were also recorded for 21 days. The percentage of hair regrowth was measured using the following equation:

$$\mathrm{Hair}\;\mathrm{regrowth}\;\left(\%\right)=\frac{\mathrm{Sample}\;\mathrm{hair}\;\mathrm{length}\;\left(\mathrm{mm}\right)-\;\mathrm{Control}\;\mathrm{hair}\;\mathrm{length}\;\left(\mathrm{mm}\right)}{\mathrm{Sample}\;\mathrm{hair}\;\mathrm{length}\;\left(\mathrm{mm}\right)}\times 100$$

with the sample hair length expressed as the mean length ± SEM of 10 hairs randomly plucked from the shaved area [56,57].

### 4.7. Statistical Analysis

All results are expressed as the mean ± SEM of experiments run in triplicate. Two-way ANOVA (followed by Tukey’s test) was used to analyse the statistical significance of the data obtained in the hair-growth promoting assay, with *p* < 0.05 considered significantly different in comparison with the control groups. One-way ANOVA (followed by Dunnett’s test) was used to analyse the statistical significance of the data obtained in the anti-inflammatory and thrombolytic assays, with *** *p* < 0.001 considered significantly different in comparison with the control groups. All statistical analyses were performed using GraphPad Prism version 8.0 (GraphPad Software Inc., San Diego, CA, USA).

### 4.8. Molecular Docking Study

#### 4.8.1. Protein Preparation

The three-dimensional crystal structure of PGD_2_ synthase (PDB ID: 2VD1) was downloaded from the RCSB Protein Data Bank (https://www.rcsb.org/, accessed on 22 April 2021). The Protein Preparation Wizard Maestro v.11.1. was used for the preparation of structure, refining, optimisation, and water removal. The OPLS3 force field was used for the energy minimization. The heavy atom molecules root-mean-square-deviation (RMSD) was set to 0.30 Å.

#### 4.8.2. Ligand Preparation

Sixteen phytochemicals, namely palmitic acid, farnesol, solanesol, phthalic acid, *n*-tricosane, *n*-tetracosane, *n*-heptacosane, *n*-tetratriacontane, *n*-octadecane, *n*-eicosane, *n*-heptadecane, 1-eicosanol, 17-pentatriacontene, lycopersen, lupeol, and β-sitosterol, previously reported in a non-polar fraction of the methanol extract of *L. indica* leaves were used for the molecular docking study [7]. All compounds were downloaded from the PubChem database in .*sdf* format. Glutathione, retrieved from its complex with PDB ID: 2VD1, was used as the positive control. Ligand preparation was performed using OPLS3 force field in LigPrep to minimize the energy when obtaining 3D structures from 2D structures.

#### 4.8.3. Grid Generation and Standard Precision (SP) Ligand Docking

The grid generation for the receptor was generated using the default parameters, with a van der Waals scaling factor of 1.00 Å and a charge cutoff of 0.25 Å. A cubic box set to 14 × 14 × 14 Å (*x*, *y*, and *z* directions) was generated focusing on the center of the active site residues. Using Glide of Schrödinger–Maestro v. 11.1, standard precision (SP) flexible ligand docking was carried out with penalties specific to non-*cis/trans* amide bonds. The partial charge limit and van der Waals scaling factor for ligand atoms were chosen as 0.15 and 0.80, respectively. Each ligand with the lowest Glide value was reported as the best docked pose [58,59].

#### 4.8.4. Ligand Efficiencies and Protein–Ligand Interactions Prediction

The Schrödinger software package Prime MM-GBSA module (OPLS3) was used to determine the free energies of binding (ΔG in kcal/mol) for each ligand and the target receptor [60,61]. The ligand efficiency for each ligand was also calculated using the ratio of ΔG to the number of heavy atoms (NHA) for each ligand, (LE = −(ΔG)/NHA) [62]. BIOVIA Discovery Studio Visualizer v.4.5 (Accelrys) was used to predict the intermolecular interactions between the best docked compounds and the PGD_2_ synthase binding site.

## Figures and Tables

**Table 1:** Docking score and ligand efficiency of glutathione and 16 phytochemicals previously reported in a non-polar fraction of the methanol extract of *L. indica* leaves [7] towards PGD_2_ synthase.

Compound	Docking Score (kcal/mol)	ΔG (kcal/mol)	Ligand Efficiency (kcal/mol)
Phthalic acid	−6.078	−41.8901	3.49
Farnesol	−2.640	−49.3485	3.08
Palmitic acid	−0.801	−47.325	2.63
*n*-octadecane	+0.634	−44.7694	2.49
*n*-eicosane	+0.571	−49.127	2.46
*n*-heptadecane	+1.125	−40.9013	2.41
*n*-tricosane	−3.022	−51.5811	2.24
1-eicosanol	+0.473	−42.9662	2.05
*n*-tetracosane	−2.625	−47.9138	1.99
*n*-heptacosane	−2.645	−51.3352	1.90
β-sitosterol	−6.191	−46.952	1.57
*n*-tetratriacontane	−2.283	−52.2674	1.54
Lycopersen	−5.672	−49.1584	1.23
Solanesol	−7.133	−47.2383	1.03
17-Pentatriacontene	−0.787	−32.9982	0.94
Lupeol	−3.992	−22.024	0.71
Glutathione (positive control)	−5.278	−34.732	1.74

**Table 2:** Molecular interactions of *L. indica* phytochemicals showing the best ligand efficiencies for PGD_2_ synthase ^1^.

Ligand	Docking Score	Ligand Efficiency	Interacting Residues	Distance (Å)	Category	Type
Phthalic acid	−6.078	3.49	Arg14	2.48, 2.73	H-Bond	Conventional
			Tyr152	1.63	H-Bond	Conventional
			Trp104	4.88, 5.06	Hydrophobic	Pi-Pi Stacked
			Met99	4.70	Hydrophobic	Pi-Alkyl
			Arg14	5.16	Hydrophobic	Pi-Alkyl
Farnesol	−2.640	3.08	Thr159	2.00	H-Bond	Conventional
			Gly13	2.13	H-Bond	Conventional
			Ile155	3.08	H-Bond	C-H bond
			Tyr152	4.76	Hydrophobic	Pi-Alkyl
			Cys156	4.91	Hydrophobic	Alkyl
			Arg14	4.55	Hydrophobic	Alkyl
			Tyr8	4.54	Hydrophobic	Pi-Alkyl
			Phe9	5.11, 4.52	Hydrophobic	Pi-Alkyl
			Met11	4.64	Hydrophobic	Alkyl
			Trp104	4.33, 4.12	Hydrophobic	Pi-Alkyl
			Leu199	4.44	Hydrophobic	Alkyl
*n*-tricosane	−3.022	2.24	Ile51	5.11	Hydrophobic	Alkyl
			Tyr8	5.32	Hydrophobic	Pi-Alkyl
			Phe9	5.42	Hydrophobic	Pi-Alkyl
			Cys156	4.28	Hydrophobic	Alkyl
			Ile155	4.72	Hydrophobic	Alkyl
			Tyr152	4.16	Hydrophobic	Pi-Alkyl
*n*-tetracosane	−2.625	1.99	Lys43	4.63	Hydrophobic	Alkyl
			Tyr152	4.02	Hydrophobic	Pi-Alkyl
			Ile155	4.93	Hydrophobic	Alkyl
			Cys156	3.84	Hydrophobic	Alkyl
			Trp104	2.79	Hydrophobic	Pi-Sigma
*n*-heptacosane	−2.645	1.90	Lys43	4.01	Hydrophobic	Alkyl
			Ile155	5.23	Hydrophobic	Alkyl
			Trp104	2.80	Hydrophobic	Pi-Sigma
			Met99	4.50	Hydrophobic	Alkyl
			Cys156	4.05	Hydrophobic	Alkyl

^1^ The control had a docking score of −5.278 kcal/mol and a ligand efficiency of 1.74.

## Data Availability

Available data are presented in the manuscript.

## References

[1] Burkill I.H. (1966). A Dictionary of the Economic Products of the Malay Peninsula.

[2] Lattif A.G., Omar I.M., Said I.M., Kadri A. (1984). A multi-variate approach to the study of medicinal plants in Malaysia. J. Singap. Natl. Acad. Sci..

[3] Chatterjee A., Pakrashi S.C. (1991). Treatise on Indian Medicinal Plants.

[4] Bourdy G., Walter A. (1992). Maternity and medicinal plants in Vanuatu. I. The cycle of reproduction. J. Ethnopharmacol..

[5] Prajapati N.D., Purohit S.S., Sharma A.K., Kumar T. (2003). A Handbook of Medicinal Plants: A Complete Source Book.

[6] Khare C.P. (2008). Indian Medicinal Plants: An Illustrated Dictionary.

[7] Srinivasan G.V., Ranjith C., Vijayan K.K. (2008). Identification of chemical compounds from the leaves of *Leea indica*. Acta Pharm..

[8] Singh D., Siew Y.-Y., Chong T.-I., Yew H.-C., Ho S.S., Lim C.S., Tan W.-X., Neo S.-Y., Koh H.-L. (2019). Identification of Phytoconstituents in *Leea indica* (Burm. F.) Merr. Leaves by High Performance Liquid Chromatography Micro Time-of-Flight Mass Spectrometry. Molecules.

[9] Wong Y.H., Abdul Kadir H., Ling S.K. (2012). Bioassay-Guided Isolation of Cytotoxic Cycloartane Triterpenoid Glycosides from the Traditionally Used Medicinal Plant *Leea indica*. Evid. Based Complement Altern. Med..

[10] Raihan M.O., Habib M.R., Brishti A., Rahman M.M., Saleheen M.M., Manna M. (2011). Sedative and anxiolytic effects of the methanolic extract of *Leea indica* (Burm. f.) Merr. leaf. Drug Discov..

[11] Mahboob T., Nawaz M., de Lourdes Pereira M., Tian-Chye T., Samudi C., Sekaran S.D., Wiart C., Nissapatorn V. (2020). PLGA nanoparticles loaded with Gallic acid- a constituent of *Leea indica* against *Acanthamoeba triangularis*. Sci. Rep..

[12] Ado M.A., Abas F., Mohammed A.S., Ghazali H.M. (2013). Anti-and pro-lipase activity of selected medicinal, herbal and aquatic plants, and structure elucidation of an anti-lipase compound. Molecules.

[13] Saha K., Lajis N.H., Israf D.A., Hamzah A.S., Khozirah S., Khamis S., Syahida A. (2004). Evaluation of antioxidant and nitric oxide inhibitory activities of selected Malaysian medicinal plants. J. Ethnopharmacol..

[14] Nurhanan M.Y., Asiah O., Ilham M.A.M., Syarifah M.M.S., Norhayati I., Sahira H.L. (2008). Anti-proliferative activities of 32 Malaysian plant species in breast cancer cell lines. J. Trop. Sci..

[15] Rahman M.A., Sultana R., Bin Emran T., Islam M.S., Rahman M.A., Chakma J.S., Rashid H.-u., Hasan C.M.M. (2013). Effects of organic extracts of six Bangladeshi plants on in vitro thrombolysis and cytotoxicity. BMC Complement Altern. Med..

[16] Reddy N.S., Navanesan S., Sinniah S.K., Wahab N.A., Sim K.S. (2012). Phenolic content, antioxidant effect and cytotoxic activity of *Leea indica* leaves. BMC Complement Altern. Med..

[17] Wong Y.H., Abdul Kadir H. (2011). *Leea indica* Ethyl Acetate Fraction Induces Growth-Inhibitory Effect in Various Cancer Cell Lines and Apoptosis in Ca Ski Human Cervical Epidermoid Carcinoma Cells. Evid. Based Complement Altern. Med..

[18] Wong Y.H., Kadir H.A. (2012). Induction of Mitochondria-Mediated Apoptosis in Ca Ski Human Cervical Cancer Cells Triggered by Mollic Acid Arabinoside Isolated from *Leea indica*. Evid. Based Complement Altern. Med..

[19] Ghagane S.C., Puranik S.I., Kumbar V.M., Nerli R.B., Jalalpure S.S., Hiremath M.B., Neelagund S., Aladakatti R. (2017). In vitro antioxidant and anticancer activity of Leea indica leaf extracts on human prostate cancer cell lines. Integr. Med. Res..

[20] Kekuda P.T.R., Raghavendra H.L., BharadwajNa A.S. (2018). Traditional uses, chemistry and pharmacological activities of *Leea indica* (Burm. f.) Merr. (Vitaceae): A comprehensive review. Int. J. Green Pharm..

[21] Dalu D., Duggirala S., Akarapu S. (2014). Anti-hyperglycemic and hypolipidemic activity of *Leea indica*. Int. J. Bioassay.

[22] Siew Y.-Y., Yew H.-C., Neo S.-Y., Seow S.-V., Lew S.-M., Lim S.-W., Lim C.S.E.-S., Ng Y.-C., Seetoh W.-G., Ali A. (2019). Evaluation of anti-proliferative activity of medicinal plants used in Asian Traditional Medicine to treat cancer. J. Ethnopharmacol..

[23] Tareq S., Ibrahim M., Shahadat S., Chowdhury M.U., Jakaria M. (2017). Comparative anti-dirrhoeal and antimicrobial activities of methanol extract of *Leea indica* (Burm. F.) Merr. and *Leea macrophylla* Roxb. Ex. Hornem (Fam. Vitaceae) and four Bangladeshi market preparations. Der. Pharma. Chem..

[24] Patel B., Patel J., Shah S. (2016). Effect of single and combinational herbal formulation in alloxan induced hyperglycemia. Der. Pharm. Lett..

[25] Sulistyaningsih E., Amalia T.Y., Kartikasari R. (2017). Antioxidant and antimalarial activity of *Leea indica* leaf extract against malaria-mice model. J. Appl. Pharm. Sci..

[26] Sreedhanya S., Athira A., Pushpalatha E. (2017). Larvicidal and repellent efficacy of some of the weed plant extracts against *Culex quinquefasciatus* Say. J. Adv. Lab. Res. Biol..

[27] Emran T.B., Rahman M.A., Hosen S.Z., Rahman M.M., Islam A.M.T., Chowdhury M.A.U., Uddin M.E. (2012). Analgesic activity of *Leea indica* (Burm.f.) Merr. Phytopharmacology.

[28] Fong P., Tong H.H.Y., Ng K.H., Lao C.K., Chong C.I., Chao C.M. (2015). In silico prediction of prostaglandin D2 synthase inhibitors from herbal constituents for the treatment of hair loss. J. Ethnopharmacol..

[29] Yamamoto J., Yamada K., Naemura A., Yamashita T., Arai R. (2005). Testing various herbs for antithrombotic effect. Nutrition.

[30] Rambwawasvika H., Dzomba P., Gwatidzo L. (2019). Hair Growth Promoting Effect of Dicerocaryum senecioides Phytochemicals. Int. J. Med. Chem..

[31] Blume-Peytavi U., Whiting D.A., Trüeb R.M. (2008). Hair Growth and Disorders.

[32] Geyfman M., Plikus M.V., Treffeisen E., Andersen B., Paus R. (2015). Resting no more: Re-defining telogen, the maintenance stage of the hair-growth cycle. Biol. Rev..

[33] Hsu Y.-C., Li L., Fuchs E. (2014). Emerging interactions between skin stem cells and their niches. Nat. Med..

[34] Lee B.H., Lee J.S., Kim Y.C. (2016). Hair growth-promoting effects of lavender oil in C57BL/6 mice. Toxicol. Res..

[35] Husain A., Khan M.S., Hasan S.M., Alam M.M. (2005). 2-Arylidene-4-(4-phenoxy-phenyl)but-3-en-4-olides: Synthesis, reactions and biological activity. Eur. J. Med. Chem..

[36] Meidan V.M., Touitou E. (2001). Treatments for androgenetic alopecia and alopecia areata. Drugs.

[37] Bergstrom K.G. (2011). What’s new in androgenetic alopecia: Approvals, long-term safety data, cancer risk and treatment options for women. J. Drugs Derm..

[38] Varothai S., Bergfeld W.F. (2014). Androgenetic alopecia: An evidence-based treatment update. Am. J. Clin. Derm..

[39] Suchonwanit P., Thammarucha S., Leerunyakul K. (2019). Minoxidil and its use in hair disorders: A review. Drug Des. Devel..

[40] Rossi A., Cantisani C., Melis L., Iorio A., Scali E., Calvieri S. (2012). Minoxidil use in dermatology, side effects and recent patents. Recent Pat Inflamm. Allergy Drug Discov..

[41] Randolph M., Tosti A. (2020). Oral minoxidil treatment for hair loss: A review of efficacy and safety. J. Am. Acad. Derm..

[42] Garza L.A., Liu Y., Yang Z., Alagesan B., Lawson J.A., Norberg S.M., Loy D.E., Zhao T., Blatt H.B., Stanton D.C. (2012). Prostaglandin D2 Inhibits Hair Growth and Is Elevated in Bald Scalp of Men with Androgenetic Alopecia. Sci. Transl. Med..

[43] Nieves A., Garza L.A. (2014). Does prostaglandin D2 hold the cure to male pattern baldness?. Exp. Derm..

[44] Hohwy M., Spadola L., Lundquist B., Hawtin P., Dahmén J., Groth-Clausen I., Nilsson E., Persdotter S., von Wachenfeldt K., Folmer R.H.A. (2008). Novel Prostaglandin D Synthase Inhibitors Generated by Fragment-Based Drug Design. J. Med. Chem..

[45] Kanaoka Y., Ago H., Inagaki E., Nanayama T., Miyano M., Kikuno R., Fujii Y., Eguchi N., Toh H., Urade Y. (1997). Cloning and crystal structure of hematopoietic prostaglandin D synthase. Cell.

[46] Takaya D., Inaka K., Omura A., Takenuki K., Kawanishi M., Yabuki Y., Nakagawa Y., Tsuganezawa K., Ogawa N., Watanabe C. (2018). Characterization of crystal water molecules in a high-affinity inhibitor and hematopoietic prostaglandin D synthase complex by interaction energy studies. Bioorg. Med. Chem..

[47] Lee I.K., Lee B.-M. (2011). Toxicological characterization of phthalic acid. Toxicol. Res..

[48] Emran T.B., Rahman M.A., Hosen S.M., Khanam U.H., Saha D. (2012). Antioxidant, cytotoxic and phytochemical properties of the ethanol extract of *Leea indica* leaf. J. Pharm. Res..

[49] Park W.-S., Lee C.-H., Lee B.-G., Chang I.-S. (2003). The extract of *Thujae occidentalis* semen inhibited 5α-reductase and androchronogenetic alopecia of B6CBAF1/j hybrid mouse. J. Derm. Sci..

[50] Shen Y.L., Li X.Q., Pan R.R., Yue W., Zhang L.J., Zhang H. (2018). Medicinal Plants for the Treatment of Hair Loss and the Suggested Mechanisms. Curr. Pharm. Des..

[51] Dhariwala M.Y., Ravikumar P. (2019). An overview of herbal alternatives in androgenetic alopecia. J. Cosmet. Derm..

[52] Emran T.B., Rahman M.A., Uddin M.M.N., Rahman M.M., Uddin M.Z., Dash R., Layzu C. (2015). Effects of organic extracts and their different fractions of five Bangladeshi plants on in vitro thrombolysis. BMC Complement Altern. Med..

[53] Babar Z.M., Jaswir I., Tareq A.M., Ali Reza A.S.M., Azizi W.M., Hafidz M., Ahfter F., Hasan M., Farhad S., Uddin M.M.R. (2019). In vivo anxiolytic and in vitro anti-inflammatory activities of water-soluble extract (WSE) of *Nigella sativa* (L.) seeds. Nat. Prod. Res..

[54] Uddin M.J., Ansari P., Rahman M.M., Mamun A., Islam M., Hazrat M., Reza A.S.M.A. (2016). Anti-inflammatory, anti-diarrheal, thrombolytic and cytotoxic activities of an ornamental medicinal plant: Persicaria orientalis. J. Basic Clin. Physiol. Pharm..

[55] Prasad S., Kashyap R.S., Deopujari J.Y., Purohit H.J., Taori G.M., Daginawala H.F. (2006). Development of an in vitro model to study clot lysis activity of thrombolytic drugs. Thromb. J..

[56] Yoon J.I., Al-Reza S.M., Kang S.C. (2010). Hair growth-promoting effect of *Zizyphus jujuba* essential oil. Food Chem. Toxicol..

[57] Zhang N.-N., Park D.K., Park H.-J. (2013). Hair growth-promoting activity of hot water extract of *Thuja Orient*. BMC Complement Altern. Med..

[58] Friesner R.A., Banks J.L., Murphy R.B., Halgren T.A., Klicic J.J., Mainz D.T., Repasky M.P., Knoll E.H., Shelley M., Perry J.K. (2004). Glide: A new approach for rapid, accurate docking and scoring. 1. Method and assessment of docking accuracy. J. Med. Chem..

[59] Friesner R.A., Murphy R.B., Repasky M.P., Frye L.L., Greenwood J.R., Halgren T.A., Sanschagrin P.C., Mainz D.T. (2006). Extra precision glide: Docking and scoring incorporating a model of hydrophobic enclosure for protein-ligand complexes. J. Med. Chem..

[60] Lionta E., Spyrou G., Vassilatis D.K., Cournia Z. (2014). Structure-based virtual screening for drug discovery: Principles, applications and recent advances. Curr. Top. Med. Chem..

[61] Chen F., Liu H., Sun H., Pan P., Li Y., Li D., Hou T. (2016). Assessing the performance of the MM/PBSA and MM/GBSA methods. 6. Capability to predict protein–protein binding free energies and re-rank binding poses generated by protein–protein docking. Phys. Chem. Chem. Phys..

[62] García-Sosa A.T., Hetényi C., Maran U. (2010). Drug efficiency indices for improvement of molecular docking scoring functions. J. Comput. Chem..

